# A Flow Cytometry-Based Assay for the Measurement of Total Complement Activity in the Serum and Clinical Practice

**DOI:** 10.1155/2022/4532511

**Published:** 2022-01-10

**Authors:** Xuewei Ding, Shijun Li, Hui Liu

**Affiliations:** College of Medical Laboratory, Dalian Medical University, Dalian 116044, China

## Abstract

**Objective:**

To develop a novel sensitive and accurate assay suitable for high-volume testing of the total complement activity in the serum for clinical laboratories.

**Methods:**

The total complement activity (TCA) to be measured was quantified by detecting the number of fragments produced by erythrocyte lysis and the erythrocyte fragmentation index (EFI), indicating TCA. EFI = *M* × *M*2/(*M*1 + *M*2), where *M* is the number of erythrocyte fragments (removed from the background), *M*1 is the number of unagglutinated red cells, *M*2 is the number of agglutinated red cell groups, and *M*2/(*M*1 + *M*2) is the agglutination coefficient indicating the degree of erythrocyte agglutination. Mild changes in hemolysin and erythrocyte concentrations were made to optimize the testing conditions. The same serum samples were tested for 10 consecutive days to determine the stability of the experimental results. Serum EFI was detected in both nephrotic syndrome patients and healthy subjects.

**Results:**

There was a linear relationship between hemolysin and erythrocyte agglutination (*r* = 0.999, *P* < 0.001). A good linear relationship existed between EFI and TCA (*r* = 0.991, *P* < 0.001). The results were not affected by slight fluctuations in the concentrations of hemolysin or erythrocytes. The interbatch CV = 8.6% of the test results showed good stability. There was a significant difference in the EFI between nephrotic syndrome patients and healthy individuals, *P* < 0.001, and EFI was reduced in nephrotic syndrome patients compared to healthy individuals.

**Conclusion:**

The flow cytometry-based assay for TCA was sensitive and accurate and had potential value for clinical application.

## 1. Introduction

The complement system consists of more than 30 components, which are widely present in normal human or vertebrate serum, tissue fluid, and cell membrane surfaces. Most components of the complement system exist in an inactive form and are only activated for biological activity. The complement system can be activated through three pathways: (1) the classical pathway, (2) the lectin pathway, and (3) the bypass pathway. Activators of the classical pathway are mainly antigen-antibody complexes, whereas activation of the classical pathway is dependent on specific antibody production [1].

The complement system plays a role in innate and adaptive immunity and is also involved in various physiological activities, including hemostasis [2], neuroprotection, synaptic pruning, and organ development [3], as well as a range of pathological activities (e.g., thrombophilia, autoimmune diseases, schizophrenia [4], allograft rejection, graft-versus-host disease, and cancer [5]). Recent findings have also confirmed that the complement system functions as a means of cellular communication in the extravascular and interstitial tissue spaces [5]. Determination of the total serum complement activity and the level of the individual components of the complement system and their activity allows for an evaluation of the status of the body's complement system and immune function. This can be used for the auxiliary diagnosis, course, and efficacy of systemic lupus erythematosus, chronic hepatitis [6], and some cancers, but is also important for the study of disease pathogenesis, other tests involving complement, and the study of complement function.

There are currently two main areas of clinical laboratory testing for the complement system: (1) testing for total complement activity (TCA) and (2) testing for individual components of the complement system [7]. The techniques used in clinical laboratories for the detection of individual components of the complement system are more mature and can be performed qualitatively, quantitatively, and automatically through various techniques, including immunoturbidimetric assays and enzyme-linked immunosorbent assays. Moreover, the clinical laboratory results for individual components of the complement system are more accurate and reproducible. Furthermore, changes in the individual complement components do not fully reflect the function of the complement system.

The determination of TCA provides a more realistic overview of the function of the body's complement system and can also be used as a basis for the detection of individual components of the complement system. The main methods used for the determination of TCA include the CP-CH50 method [8], the AP-CH50 method [9], the single tube titration method [10], and the plate-hemolysis method [11]. The CP-CH50 method is widely used in clinical laboratories, which uses anti-sheep erythrocyte antibody-sensitized sheep erythrocytes (SRBC) as the activator and indicator system to activate the classical complement pathway. In contrast, SRBC hemolysis is used to indicate the TCA of serum samples. However, the CP-CH50 method is a manual, complex, semiquantitative assay with long lead times. Moreover, fresh SRBC are difficult to collect, transfer, and preserve, and their quality is not guaranteed [12]. The AP-CH50 method, the single tube titration method, and plate-hemolysis method are essentially the same as the CP-CH50 method in terms of principle and interpretation of results, but do not overcome the shortcomings of yielding unstable results.

This study was based on the CP-CH50 method. Human O-erythrocytes sensitized with anti-human O-erythrocyte antibodies were used as an indicator system and activator of the classical complement pathway. Samples were analyzed by flow cytometry as a means of observing human O-erythrocyte fragments. The aim of this study was to create a rapid, simple, highly sensitive, accurate, and reproducible method for the determination of TCA that is truly representative of the global TCA and is suitable for automated analysis. These findings will overcome the drawbacks of the CP-CH50 method, which is complex, has low sensitivity, and is unstable in intercompartmental quality assessment. Moreover, our results seek to advance the clinical application of the TCA assay and assist in the progress of complement-related research.

## 2. Materials and Methods

### 2.1. Reagents

PBS buffer was generated by adding 0.434 g magnesium chloride hexahydrate (MgCl_2_-6H_2_O), 0.033 g calcium chloride (CaCl_2_), and 11.2 g PBS powder in turn to distilled water for dissolution, followed by the continued addition of distilled water to a volumetric flask to a final volume of 1000 mL. The buffer was stored at 4°C.

To isolate 2% human O-erythrocytes, 1 mL of fresh human O-anticoagulated blood was centrifuged at 2500 rpm (1118 × *g*). The plasma was discarded, and 4 mL sterile saline was added to the remaining red cells and washed by centrifugation at 2500 rpm (1118 × *g*) for 5 min. This process was repeated twice, and the supernatant liquid was discarded. Next, 4 mL of buffer was added 2500 rpm (1118 × *g*) and washed twice by centrifugation for 5 min, until the supernatant liquid was clear and transparent. The 2% human O-erythrocytes were obtained by mixing 20 *μ*L of pressurized erythrocytes in 980 *μ*L of buffer.

Serum was obtained by collecting venous blood from healthy volunteers, leaving it to stand for 1 h at 4°C, and centrifuged at 3000 rpm (1610 × *g*) for 10 min to separate the serum for further use.

The 4 U hemolysin (anti-human O-erythrocyte antibody, trade name rabbit anti-human O-erythrocyte serum, potency 1 : 100, supplied by Zhengzhou Baiji Biotechnology Co., Ltd.) was diluted in the buffer mentioned above according to potency (this concentration unit was based on the potency of hemolysin when it caused an agglutination reaction; when the potency of hemolysin was 100, we diluted it 25 times to obtain 4 U of hemolysin).

### 2.2. Flow Cytometry

**Figure 1:**
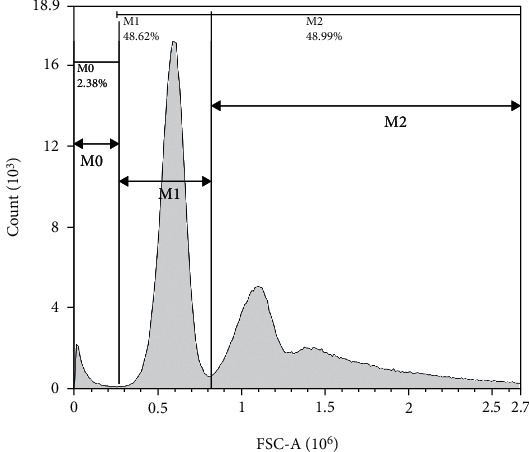
Schematic diagram of the flow cytometry setup gate. Note: *M*0 is residual hemolysin, and other impurities were in the agglutination reaction or were erythrocyte fragments containing background in the hemolysis reaction; *M*1 is unagglutinated erythrocytes; and *M*2 is agglutinated erythrocytes.

In this study, flow cytometry was combined with the CP-CH50 method. After an agglutination or hemolysis reaction, the reaction system could be divided into three main parts: (1) the erythrocyte agglutination group, (2) the unagglutinated erythrocytes, and (3) the fragmented erythrocytes; the volume of three decreased in turn (both their pulse signal FSC-A decreasing in turn). A two-dimensional histogram of the size and number of pulsed signals (FSC-A) was obtained by flow cytometry and gated from right to left for each peak segment as *M*2, *M*1, and *M*0, respectively, as shown in Figure 1. The average erythrocyte diameter in normal subjects was 7.0 *μ*m-7.6 *μ*m, with an average of 7.33 *μ*m. Flow cytometry was performed using a NovoCyte Flow Cytometer (NovoCyte2040R). This study was controlled to the extent that the instrument constraints allowed in order to better distinguish between clumped erythrocytes, unclumped erythrocytes, and fragmented erythrocytes. The sample flow diameter was 7.7 *μ*m (closest to the average erythrocyte diameter).

For reference, the flow cytometry parameters were set as follows: threshold setting, FSC‐H > 100,000; stop condition setting, 30 *μ*L; sample flow rate setting, 14 *μ*L/min; and sample flow diameter, 7.7 *μ*m. We obtained 2D histograms with FSC-A on the *x*-axis and count on the *y*-axis.

The above reagent and instrument settings were used unless otherwise specified.

### 2.3. Detection of Human O-Erythrocyte Hemolysis Caused by Complement by Flow Cytometry

The degree of hemolysis varied with different concentrations of serum. Thus, the higher the serum concentration, the greater the degree of hemolysis and higher number of erythrocyte fragments in the reaction tube. This experiment attempted to demonstrate that complement-induced erythrocyte hemolysis could be detected both qualitatively and quantitatively by flow cytometry.

Sera were obtained from healthy volunteers and diluted as follows: original, 1 : 2, 1 : 4, 1 : 8, and 1 : 16.

The reagents were added in accordance with Table 1, and the test was carried out with reaction tube 1 as the control tube. The hemolysin in reaction tubes 2-6 was arranged from the smallest to the largest fold of the fold dilution. The individual reaction tubes were placed in a constant temperature water bath at 37°C for 30 min. At the end of the water bath incubation, the tubes were mixed thoroughly (without filtering) and assayed by flow cytometry.

### 2.4. Detection of Human O-Erythrocyte Agglutination Caused by Hemolysin by Flow Cytometry

Complement-mediated hemolysis was based on an erythrocyte agglutination reaction, which occurs in response to erythrocyte agglutination, forming hemolysin-sensitized erythrocytes (antigen-antibody complexes) prior to activation of the classical pathway. Activation of the classical complement pathway results in the formation of a membrane attack complex (MAC) that breaks down the erythrocyte membrane and leads to the formation of erythrocyte fragments. This experiment attempted to demonstrate that flow cytometry could detect human O-erythrocyte agglutination caused by hemolysin (rabbit anti-human O-erythrocyte antibody), thereby enabling the quantification of antibodies in the reaction system.

Prior to use, hemolysin was diluted to 32 U in buffer according to its potency. 32 U of hemolysin was diluted as follows: original multiple, 1 : 2, 1 : 4, 1 : 8, and 1 : 16.

The reagents were added in accordance with Table 2, and the test was carried out with reaction tube 1 as the control tube and the hemolysin in reaction tubes 2-6 were arranged from the smallest to largest dilutions. The individual reaction tubes were placed in a constant temperature water bath at 37°C for 30 min. After the incubation in the water bath, the samples were analyzed by flow cytometry.

### 2.5. Optimization of Interfering Factors and Experimental Conditions for the Erythrocyte Hemolysis Test

The flow cytometer was highly sensitive and could precisely detect the erythrocytes and hemolysin as the influencing factors in this experiment. We investigated the ability of major influencing factors to interfere with the experimental results and the effect of eliminating their interference.

The concentration of erythrocytes used for the reaction varied by ±10%, and 2%, 2.2%, and 1.8% solutions of human O-erythrocytes were configured for the test. The concentration of hemolysin for the reaction was altered by ±10% to 4 U, 4.4 U, and 3.6 U of hemolysin solution for the assay.

The reagents were added as outlined in Table 3, which shows the hemolysis reactions for each condition (reactions 1-5 were repeated five times each) and the erythrocyte hemolysis test was performed. The serum for each reaction was replaced with the same volume of buffer, and the reactions under the same conditions were repeated five times to obtain the mean values of their background and agglutination coefficients. The individual reaction tubes were placed in a constant temperature water bath at 37°C for 30 min. After incubating the samples in a water bath, they were analyzed by flow cytometry.

### 2.6. Establishment of a Flow Cytometry-Based Assay for the Detection of Total Complement Activity

This assay is aimed at demonstrating good quantitative efficacy for the ability of flow cytometry to detect erythrocyte hemolysis caused by different complement concentrations following the introduction of coagulation coefficient parameters. Thus, a preliminary flow cytometry-based assay could be established for the detection of TCA.

Sera were obtained from healthy volunteers and diluted as follows: original, 1 : 2, 1 : 4, 1 : 8, and 1 : 16. The reagents were added as described in Table 2, and the test was carried out. Reaction tube 1 was repeated five times to obtain the background and agglutination coefficients. The individual reaction tubes were placed in a constant temperature water bath at 37°C for 30 min. After incubation in a water bath, the samples were analyzed by flow cytometry. The agglutination coefficient was introduced to obtain the erythrocyte fragmentation index.

### 2.7. Comparison of the Resolution of the Erythrocyte Fragmentation Index and OD Values to Measure the Total Complement Activity

The resolution of the assay reflects its ability to detect different degrees of changes in its experimental indicators. The higher the resolution of the test method, the smaller the degree of changes in the experimental indicators that the assay can detect and increase the sensitivity of the assay [13]. This experiment sought to demonstrate the high resolution of TCA as measured by the erythrocyte fragmentation index: EFI = *M* × the mean value of agglutination coefficient. The EFI was positively correlated with TCA in the serum; the higher TCA in the serum, the greater the EFI.

Sera were obtained from healthy volunteers and diluted into a 50% concentration gradient of equal proportions (one part serum was mixed with one part buffer, one part of the mixture was removed for use, and one part of the buffer was added; the process was repeated for a series of dilutions, with a 50% difference between adjacent mixtures) and 25% concentration gradient of equal-proportion dilutions (three parts serum were mixed with one part buffer; one part of the mixture was removed for use, one part buffer was added, and the process was repeated for a series of dilutions). This procedure was repeated for a series of dilutions (with a 25% difference in concentration between adjacent mixtures), 10% concentration gradient of dilutions in equal proportions (nine sera mixed with one buffer, one buffer removed for use, one buffer was added, and the procedure was repeated for a series of dilutions with a 10% difference in the concentrations between adjacent mixtures), and four dilutions of equal proportion for each type, including the undiluted serum. There were five different concentrations of serum in each equal-proportion dilution series.

Reagents were added as described in Table 2, and the test was performed. The individual reaction tubes were placed in a constant temperature water bath at 37°C for 30 min. After incubation in a water bath, the samples were analyzed by flow cytometry. After the completion of flow cytometry, the tubes were centrifuged at 2500 rpm (1118 × *g*) for 5 min and 100 *μ*L was taken at an absorbance value of 542 using an optical density meter.

### 2.8. Test of the Stability of the Total Complement Activity Detection Results by Flow Cytometry

Sera were obtained from healthy volunteers, and the serum was separated into 10 portions (100 *μ*L each). The reagents were added as described in Table 4, and tested for 10 days for the CV.

### 2.9. Detection of Human O-Erythrocyte Hemolysis Caused by Serum Complement in Healthy Subjects and Nephrotic Syndrome Patients by Flow Cytometry

TCA was measured in healthy subjects and nephrotic syndrome patients using a flow cytometry-based assay to demonstrate the clinical laboratory value for this assay. In this study, the following clinical diagnostic indexes in the serum of clinical patients with nephrotic syndrome were used: (1) urinary protein > 3.5 g/d, (2) serum albumin lower than 30 g/L, (3) oedema, and (4) hyperlipidaemia. Among these, (1) and (2) are necessary for a diagnosis [14].

The specimens were obtained from the First Affiliated Hospital of Dalian Medical University, China. The serum of 30 patients with clinically diagnosed nephrotic syndrome was selected as the disease group. Serum samples from 30 healthy subjects with no abnormal laboratory indicators were used as the control group. Serum dates were consistent between batches in both groups. The mean ± SD of healthy subjects was 46.4 ± 15.7, and the mean ± SD of nephrotic syndrome patients was 41.5 ± 16.8. A paired *t*-test was conducted for the age of the two groups of samples (*P* = 0.266), and there was no significant difference in the age between the two groups of candidates. The control group was divided evenly between men and women.

### 2.10. Statistical Analysis

The results were plotted as a linear graph for each dilution and or logarithm of each dilution against the processed values of the flow cytometry counting results processing value. A multivariate analysis was used to evaluate the influence of erythrocytes and changes to the antibody concentration on the test results. Different concentrations of erythrocytes and hemolysin were considered as the independent variables, and pieces of the erythrocyte number (*M*) value and index of erythrocyte debris (EFI), respectively, were used as the dependent variables. These variables were used to proceed with analysis of regression to assess the impact of changes to erythrocytes and antibody concentrations on the detection results of the experiment and optimization. A linear regression analysis was used to detect the resolution of the human O-erythrocyte hemolysis results caused by complement using flow cytometry and optical densitometry. The stability of the results of the complement-induced human O-erythrocyte hemolysis assay by flow cytometry was analyzed using the coefficient of variation. A paired *t-*test with a standard deviation of the mean was used to assess the differences between the two groups of samples from healthy individuals and nephrotic syndrome patients. SPSS statistical software was used for these analyses.

## 3. Results

### 3.1. Experimental Results of Complement-Induced Hemolysis of Human O-Erythrocytes Detected by Flow Cytometry

**Figure 2:**
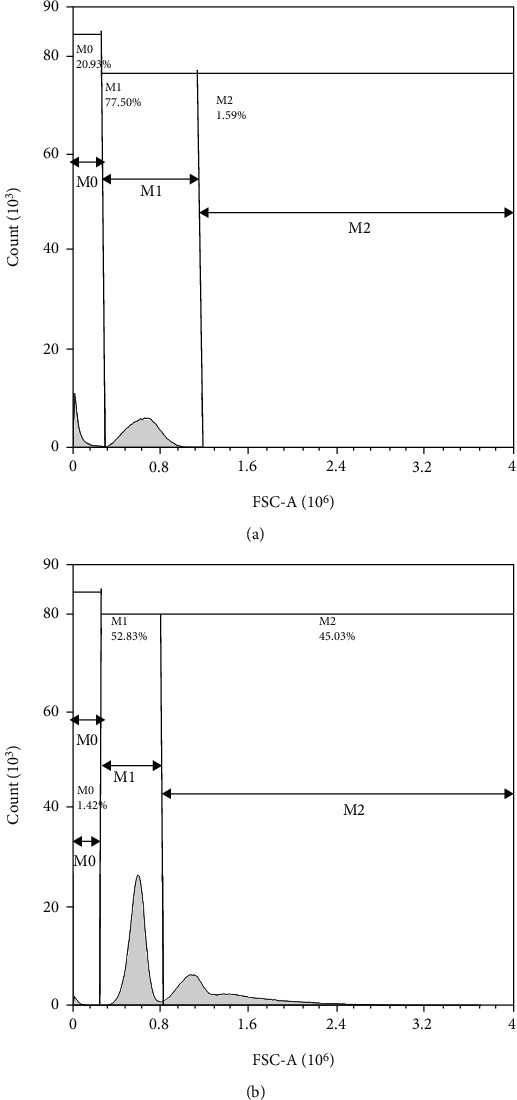
Experimental flow cytometry results of human O-erythrocyte hemolysis induced by hemolysin. Note: (a) is the result of the hemolytic reaction of 4 U hemolysin, and (b) is the result of the agglutination reaction of the control; *M*0 is red cell fragments, *M*1 is unagglutinated red cells, and *M*2 is agglutinated red cells.

Flow cytometry was performed to detect complement-induced human O-type erythrocyte hemolysis.  Figure 2 presents reaction 2 (the undiluted serum reaction) and reaction 1 (blank control reaction) shown. The statistics for the *M*0 count, *M*1 Abs.count, and *M*2 Abs.count are presented in Table 5.

Table 5 also shows that the count value of the first *M*0 peak decreases with an increasing dilution in each tube, whereas the second and third peaks for *M*1 and *M*2 exhibit an increasing trend. Therefore, the first peak for *M*0 mainly consisted of erythrocyte fragments, whereas the second and third peaks for *M*1 and *M*2 were comprised of unagglutinated and agglutinated erythrocytes, respectively. The count value of the first peak of the reaction in tube 1 was used as the background, and the difference between the count value of the first peak of the remaining reaction tubes and the count value of the first peak of reaction tube 1 was the actual measured value of the hemolysis result: *M*, its calculation formula is presented in

(1) $$\begin{array}{c} M=\text{response value}–\text{backgroundvalue}. \end{array}$$

**Figure 3:**
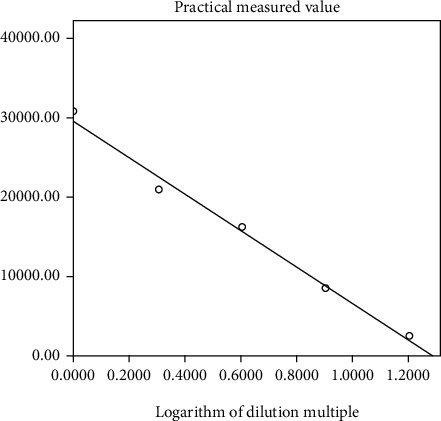
Correlation of hemolytic reactions due to serum multiplicative dilution.

The logarithm of the dilution multiplier was used as the horizontal coordinate and the measured value *M* was used as the vertical coordinate for the statistical plot, and the results are shown in Figure 3.

Based on the results listed in Table 5, for each reaction *M*0, *r* for linearity of the statistical analysis was 0.991 (*P* < 0.001). Therefore, the measured value *M* has a good linear relationship with the logarithm of the dilution. Thus, flow cytometry can quantify human O-erythrocyte hemolysis caused by complement.

### 3.2. Experimental Results of Human O-Erythrocyte Agglutination Caused by Hemolysin as Detected by Flow Cytometry

**Figure 4:**
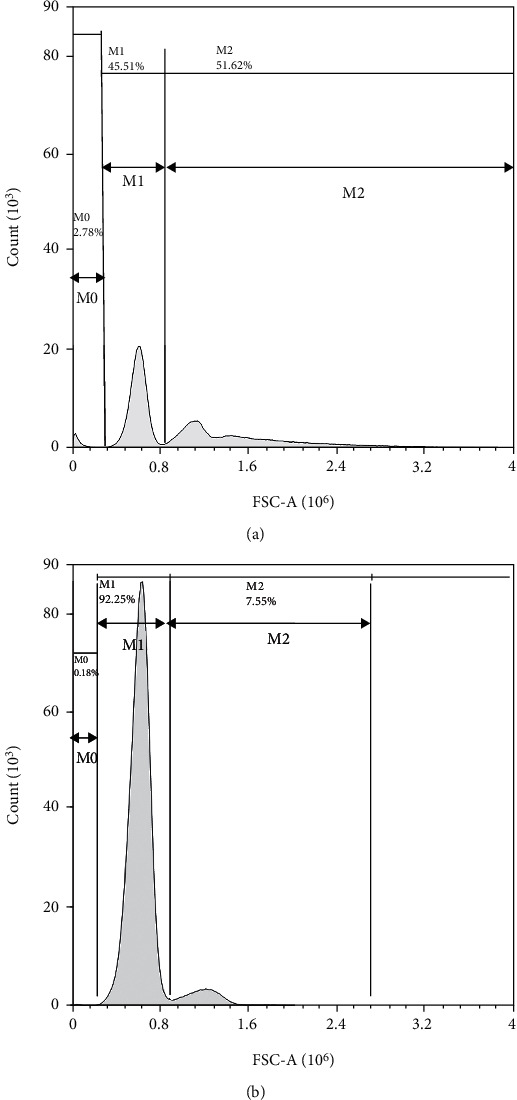
Experimental flow cytometry results of hemolysin-induced agglutination of human O-erythrocytes. Note: (a) is the result of the agglutination reaction with 4 U of hemolysin, and (b) is the result of the reaction with the blank control; *M*0 is the remaining hemolysin and other impurities, *M*1 is the unagglutinated erythrocytes, and *M*2 is the agglutinated erythrocytes.

Flow cytometry was used to detect hemolysin-induced human O-erythrocyte agglutination, as shown in Figure 4. (reaction 5, the 4 U hemolysin reaction; reaction 1, the blank control reaction).

Figures 4(a) and 4(b) show that the flow cytometric analysis of the tubes in which hemolysin was present (tubes in which agglutination occurred) differed significantly from those in which hemolysin was not present (tubes in which agglutination did not occur). Such differences were characterized as an increase in the number of peaks (i.e., the appearance of the first peak, *M*0) and a change in the peak and peak distance (i.e., a decrease in the second peak, *M*1, and an increase in the third peak, *M*2, or an increase in width). During the agglutination reaction, erythrocytes agglutinate with each other due to hemolysin, resulting in a decrease in the total number of erythrocytes and an increase in the number of agglutinated erythrocyte groups. Therefore, the second peak was in the region of unagglutinated erythrocytes, the third peak was in the region of agglutinated erythrocytes, and the first peak was in the region of impurities (e.g., residual hemolysin from the experiment and a small number of fragments formed from broken erythrocytes during the reaction). The results of the reactions without the addition of hemolysin are bimodal, with the left-hand side of the peak being a dummy signal generated by the detection channel for normal erythrocytes and/or small volumes of erythrocytes. Moreover, the right-hand side of the peak was a dummy signal generated by the detection channel for a few large volumes of erythrocytes and/or the concave surfaces of erythrocytes. The calculated *M*0 count, *M*1 Abs.count, *M*2 Abs.count, and *M*2/(*M*1 + *M*2) values are presented in Table 6.

**Figure 5:**
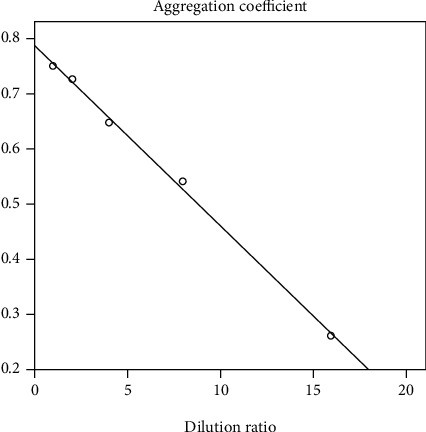
Correlation of agglutination reactions caused by hemolysin multiplier dilution.

Table 6 shows that the *M*0 count value of the first peak decreased as the concentration units of hemolysin decreased. In addition, the *M*0 count value of the first peak for the 4 U hemolysin reaction and the 2 U hemolysin reaction was the closest to the *M*0 count value of the first peak of the background reaction. Due to the differences between the *M*1 Abs.count value of the second peak of the 4 U hemolysin, the reaction and *M*1 Abs.count value of the second peak in the control reaction were more pronounced than the difference between the M1 Abs.count value of the second peak of the 2 U hemolysin reaction and the background reaction. The 4 U hemolysin was used in subsequent assays unless indicated otherwise. The ratio *M*2/(*M*1 + *M*2) (which the authors refer to as the coagulation coefficient and whose formula is provided in Equation (2)) was statistically plotted using the dilution multiple as the horizontal coordinate and the *M*2/(*M*1 + *M*2) ratio as the vertical coordinate. The results are shown in Figure 5.

(2) $$\begin{array}{c} \text{Coagulation coefficient}=\frac{M2}{\left(M1+M2\right)}. \end{array}$$

Based on the results of th agglutination coefficients for each reaction in Table 6, the linearity was statistically analyzed at *r* = 0.999, *P* < 0.001. It can be concluded that the agglutination coefficients had a good linear relationship with the dilution times. Thus, flow cytometry could be used to qualitatively and quantitatively analyze human O-erythrocyte agglutination caused by hemolysin within a certain range.

### 3.3. Establishment and Interference Experiments with a Total Complement Activity Assay by Flow Cytometry

The interference factors for the erythrocyte hemolysis test caused by complement and optimization of experimental conditions were detected by flow cytometry. The experimental results were obtained by flow cytometry, and *M* of each reaction was obtained (see Table 7 for *M* of each reaction). The product of each *M* in the reaction and the average agglutination coefficient of the corresponding five background reactions is denoted as the erythrocyte fragmentation index (EFI) (the EFI for each reaction is shown in Table 7, and the calculation method is shown in Formula (3)), for which EFI = *M* × the mean value of agglutination coefficient. The EFI was positively correlated with TCA in the serum; the higher TCA in the serum, the greater the EFI. Therefore, EFI can be used to indicate TCA.

(3) $$\begin{array}{c} \text{EFI}=M*\frac{M2}{\left(M1+M2\right)}. \end{array}$$

Note: *M* = response value − background value; *M*2/(*M*1 + *M*2) is the coagulation coefficient.

A multifactor analysis was used to obtain a significant *P* value of 0.202 for erythrocytes and significant *P* < 0.001 for hemolysin. Therefore, the test result for *M* in this assay was primarily affected by the factor of hemolysin; after introducing the agglutination coefficient as a parameter, the significant *P* value in this experiment with erythrocytes was 0.709, and the significant *P* value of hemolysin was 0.727. Therefore, there was good anti-interference ability for the detection of human O-type erythrocyte hemolysis caused by complement using flow cytometry.

### 3.4. Linear Observation of the Total Complement Activity Based on Flow Cytometry

**Figure 6:**
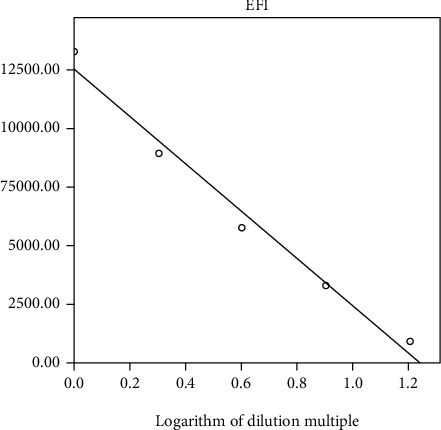
Linear model for the flow cytometry-based assay for TCA.

Flow cytometry was performed to detect TCA. After the experimental results were obtained by flow cytometry, *M*, EFI, and the average values of the agglutination coefficient for each of the reaction test results were counted (Table 8). The logarithm of the dilution was used as the horizontal coordinate and EFI was used as the vertical coordinate for Figure 6.

The statistical analysis showed a linearity of *r* = 0.991, *P* < 0.001. Therefore, it can be concluded that the introduction of the agglutination coefficient resulted in a good linearity of the experimental results for the detection of erythrocyte hemolysis caused by different complement concentrations using flow cytometry. This led to the establishment of a preliminary flow cytometry-based assay for the detection of TCA.

### 3.5. Experimental Results Comparing the Resolution of TCA Measured by EFI and OD Values as Test Indicators

To compare the resolution of TCA as measured by EFI and OD, flow cytometry was performed to calculate the EFI. The difference between the OD of each hemolysis reaction (reaction tubes 1-5) and the OD of the corresponding background reaction (reaction tube 6) (background OD) was the measured OD (i.e., actual OD = reaction OD − background OD) as listed in Table 9.

A linear fit was performed using the percentage of the actual concentration as the independent variable. The EFI and the actual measured OD value were used as the dependent variables. The results in Table 9 show that flow cytometry had a higher resolution and greater sensitivity for detecting the hemolysis of human O-erythrocyte caused by complement.

### 3.6. Experimental Results on the Stability of the Assay Results for Complement-Induced Hemolysis of Human O-Erythrocytes by Flow Cytometry

The TCA stability test was conducted based on flow cytometry. After the results were obtained by flow cytometry, EFI and the mean values of the agglutination coefficient of each reaction were calculated for each day, as shown in the results in Table 10.

Because the 10-day CV of the EFI was 8.6%, the results of the flow cytometry assay for complement-induced human O-erythrocyte hemolysis had good stability.

### 3.7. Experimental Results of Human O-Erythrocyte Hemolysis Induced by Serum Complement in Healthy Subjects and Nephrotic Syndrome Patients Detected by Flow Cytometry

TCA was measured in healthy subjects and nephrotic syndrome patients using a flow cytometry-based assay. The results of the assay are shown in Table 11.

The paired *t*-test for the EFI of the two samples was *P* < 0.001. The mean EFI (10455.5) in healthy individuals was greater than the mean EFI (8778.8) in patients with nephrotic syndrome, indicating a higher TCA in healthy individuals than in nephrotic syndrome patients. This finding was consistent with the conclusions reached in previous studies using the CP-CH50 method. This suggested that a flow cytometry-based assay for TCA had some value for clinical laboratory application.

## 4. Discussion

In this study, a series of experiments were conducted to establish a flow cytometry-based assay for TCA using human O-erythrocytes as the indicator system. Human O-erythrocytes were found to be easily accessible and of reliable quality, resulting in stable and accurate experimental results [15]. This assay was based on the CP-CH50 method, which was premised on the principle of using O-erythrocytes as an antigen and hemolysin (rabbit anti-O-erythrocyte antibody) as an antibody. Together, these components formed an antigen-antibody complex that activated the classical complement pathway, forming a MAC on the surface of the erythrocyte, which perforated the surface of the erythrocyte membrane to lyse the erythrocytes. These erythrocyte fragments were then detected and counted by flow cytometry. The number of erythrocyte fragments was positively correlated with TCA. The flow cytometry-based assay for TCA and the CP-CH50 method differed in that the former used erythrocyte fragmentation as an experimental observation, converting the number of erythrocyte fragments into an EFI to indicate TCA. In contrast, the latter essentially used the OD of hemoglobin in the solution as an observable, converting the OD value into a CH50 value. Thus, since erythrocyte fragmentation represents a more direct experimental observation than OD, EFI is a more realistic and reliable indicator of TCA.

In this study, the number of erythrocyte fragments exhibited a good linear relationship with the logarithm of the dilution by increasing the amount of serum, and the number of erythrocyte fragments showed a positive correlation with the logarithm of the dilution. The flow cytometry-based assay for TCA was essentially a hemolytic reaction based on an erythrocyte agglutination reaction. In the context of quantifying complement in the serum, the degree of erythrocyte agglutination could have impacted on the results. Therefore, this experiment was first developed to determine the degree of erythrocyte agglutination by flow cytometry [16] (i.e., to quantify the addition of erythrocytes and hemolysin). In this process, the agglutination coefficient, a new detection index for human O-type erythrocyte agglutination (3.2 for the calculation method), was established in this study. The agglutination coefficient reflected the actual agglutination capacity of the erythrocytes at a certain concentration and hemolysis at a specific concentration, thereby eliminating the experimental error caused by the process of resting the specimen. This can provide novel insight into the establishment of new agglutination indexes.

Therefore, this study investigated the factors influencing the flow cytometry-based assay of TCA. There were three main reaction components in this study, serum, hemolysin, and erythrocytes. Because serum was the object of the assay, this study investigated hemolysin and erythrocytes as the objects of the influencing factors. Through a series of experiments, a multifactor analysis of the measured value of *M* found that hemolysin (*P* < 0.001) had a significant effect on the measured value of *M*. Therefore, this study correlated the measured value of *M* with the agglutination coefficient and created a test index of erythrocyte fragmentation index (EFI), EFI = *M*2/(*M*1 + *M*2) × (reaction value − background value) and *M*2/(*M*1 + *M*2) was the coagulation coefficient. After the introduction of the agglutination coefficient, a multifactorial analysis of the assay index erythrocyte fragmentation index revealed *P* values of 0.709 and 0.727 for erythrocytes and hemolysin, respectively, successfully eliminating the effect of hemolysin and erythrocytes on the experimental results (Table 7).

EFI was used as an indicator, and EFI was positively correlated with the logarithm of the dilution, indicating that the magnitude of EFI reflected the strength of TCA in the serum (Table 8; Figure 6). In this study, the EFI of the same serum was tested for 10 consecutive days with a CV = 8.6%, which suggested that the results of the flow cytometry-based assay for TCA were stable (Table 10). This method was found to have a better resolution during the course of experiments compared to the resolution obtained using OD.

Relevant clinical studies have confirmed a reduction in TCA in patients with nephrotic syndrome compared to normal reference values, and that patients in recovery may exhibit increased reactivity [17]. This method was applied to the determination of TCA in the sera of healthy individuals and nephrotic syndrome patients. A paired *t*-test of the age of the two samples (*P* = 0.266) indicated that age had no effect on the test results. The mean EFI (10455.5) in healthy subjects was greater than that of 8778.8 in nephrotic syndrome patients and the paired *t*-test of the EFI between the two groups of samples was statistically significant (*P* < 0.001). This finding indicates that a flow cytometry-based assay can effectively determine the TCA in clinical laboratory applications. In this study, a statistical analysis of the EFI of healthy individuals was performed, suggesting an EFI range of 8500-13000 as a normal reference range.

Currently, most clinical laboratories use the CP-CH50 method to determine the level of TCA in serum. The CP-CH50 method is complex to perform, has a long test period, cannot be readily stored, and does not guarantee the quality of SRBC as part of the indicator system. Furthermore, the observation of the test results of the CP-CH50 method is influenced by subjective factors, which is not conducive to an interval quality assessment of the test results. In this study, we developed a novel method for the determination of the TCA in serum that was simple to perform, had a short test cycle, and provided stable results.

In summary, this assay provided a rapid, efficient, accurate, and sensitive assay for the detection of TCA in serum and also had the potential to facilitate a high-volume assay to detect the total complement activity present in serum in clinical laboratories. This assay may also be useful for more in-depth studies of hemolytic and agglutination reactions, as well as studies of diseases associated with complement abnormalities and function.

## Figures and Tables

**Table 1:** Reagent dosage for complement-induced human O-erythrocyte hemolysis test, detected by flow cytometry.

Reaction tube number	1	2	3	4	5	6
Buffer (*μ*L)	50	0	0	0	0	0
Hemolysin (*μ*L)	100	100	100	100	100	100
Serum (*μ*L)	0	50	50	50	50	50
2% human O-erythrocytes (*μ*L)	50	50	50	50	50	50

**Table 2:** Reagent dosage for the human O-erythrocyte agglutination test caused by hemolysin, detected by flow cytometry.

Reaction tube no.	1	2	3	4	5	6
Buffer (*μ*L)	150	50	50	50	50	50
Hemolysin (*μ*L)	0	100	100	100	100	100
2% human O-erythrocytes (*μ*L)	50	50	50	50	50	50

**Table 3:** Interference factors and reagent amounts optimized for the complement-induced erythrocyte hemolysis test, detected by flow cytometry.

	Test tube 1	Test tube 2	Test tube 3	Test tube 4	Test tube 5
Serum (*μ*L)	50	50	50	50	50
4 U hemolysin (*μ*L)	100	100	100	0	0
4.4 U hemolysin (*μ*L)	0	0	0	100	0
3.6 U hemolysin (*μ*L)	0	0	0	0	100
2% erythrocytes (*μ*L)	50	0	0	50	50
2.2% erythrocytes (*μ*L)	0	50	0	0	0
1.8% erythrocytes (*μ*L)	0	0	50	0	0

**Table 4:** Experimental system for flow cytometry-based detection of TCA.

	Control reaction	Testing reaction
Buffer (*μ*L)	50	0
Hemolysin (*μ*L)	100	100
Serum (*μ*L)	0	50
2% human O-erythrocytes (*μ*L)	50	50

Note: when the same batch of 2% human O-erythrocytes and hemolysin as the reaction reagent was used, only the control reaction needed to be repeated five times to obtain the average control and agglutination coefficient of the five experiments for all serum samples tested in that batch.

**Table 5:** Results of hemolysis reactions due to hemolysin multiplier dilution.

Reaction number	*M*0 count	*M*
1. Control	8145	0
2. 1x dilution	38895	30750
3. 2x dilution	29104	20959
4. 4x dilution	24331	16186
5. 8x dilution	16740	8595
6. 16x dilution	10605	2460

**Table 6:** Detection values for the hemolysin multiplier dilution agglutination reactions *M*0, *M*1, and *M*2.

Reaction number	*M*0 count	*M*1 Abs.count	*M*2 Abs.count	*M*2/(*M*1 + *M*2)
1. Control	4111	40916	3862	0.0862
2. 32 U hemolysin	127387	1106	3339	0.7512
3. 16 U hemolysin	58812	1971	5243	0.7268
4. 8 U hemolysin	28477	4257	7852	0.6484
5. 4 U hemolysin	14503	8333	9800	0.5405
6. 2 U hemolysin	6994	27663	9882	0.2632

**Table 7:** Multifactor analysis of the effects of erythrocytes and hemolysin on measured *M* and EFI.

*M*	Changes in erythrocyte concentration	Changes in hemolysin concentration	EFI	Changes in erythrocyte concentration	Changes in hemolysin concentration
19116.60	0	0	9193.17	0	0
16946.60	0	0	8149.62	0	0
16933.60	0	0	8143.37	0	0
17452.60	0	0	8392.96	0	0
17826.60	0	0	8572.81	0	0
19460.00	10%	0	8834.84	10%	0
18631.00	10%	0	8458.47	10%	0
19461.00	10%	0	8835.29	10%	0
19454.00	10%	0	8832.12	10%	0
20115.00	10%	0	9132.21	10%	0
16579.80	-10%	0	9070.81	-10%	0
15136.80	-10%	0	8281.34	-10%	0
16427.80	-10%	0	8987.65	-10%	0
15372.80	-10%	0	8410.46	-10%	0
15330.80	-10%	0	8387.48	-10%	0
19020.20	0	10%	8859.61	0	10%
17051.20	0	10%	7942.45	0	10%
18014.20	0	10%	8391.01	0	10%
16207.20	0	10%	7549.31	0	10%
17358.20	0	10%	8085.45	0	10%
18559.80	0	-10%	9478.49	0	-10%
15486.80	0	-10%	7909.11	0	-10%
15737.80	0	-10%	8037.29	0	-10%
15117.80	0	-10%	7720.66	0	-10%
16346.80	0	-10%	8348.31	0	-10%
Beta value	0.264	0.754	Beta value	-0.078	0.074
*T* value	1.313	5.5	*T* value	-0.378	0.354
*P* value	0.202	<0.001	*P* value	0.709	0.727

**Table 8:** Experimental results of flow cytometry-based assays for TCA.

Dilution	*M*	EFI
1x	28542	13283.45
2x	19285	8975.24
4x	12432	5785.85
8x	7066	3288.52
16x	1892	880.54

Note: mean coagulation coefficient = 0.4654.

**Table 9:** Results of resolution comparison experiments using EFI and OD as assay indicators for TCA.

Equal-proportion dilutions	50%			25%			10%		
	Dilution gradient	EFI	OD	Dilution gradient	EFI	OD	Dilution gradient	EFI	OD
	1000%	13022.630	0.273	1000%	18500.730	0.352	1000%	24188.890	0.331
	500%	8876.140	0.223	750%	14796.720	0.323	900%	22465.690	0.332
	250%	6854.770	0.219	563%	12285.720	0.307	810%	21233.090	0.333
	125%	3639.980	0.112	422%	8172.260	0.312	729%	17937.660	0.327
	63%	1041.810	0.036	316%	5555.190	0.199	656%	17282.400	0.311
*R*	—	0.952	0.825	—	0.986	0.797	—	0.980	0.742
*P*	—	0.012	0.086	—	0.002	0.106	—	0.003	0.151

**Table 10:** Experimental results based on the stability of TCA assay results by flow cytometry.

No. of days	EFI	Mean ± SD	CV
1	7935.25		
2	8431.86		
3	8036.21		
4	7967.51		
5	8815.51	7974.67 ± 683.24	8.6%
6	8039.72		
7	7518.41		
8	7531.41		
9	6588.50		
10	8928.28		

**Table 11:** EFI in healthy participants and patients with nephrotic syndrome.

Group	Mean	SD	*T* value	*P* value
Nephrotic syndrome patients	8778.8	2052.4	3.748	<0.001
Healthy subjects	10455.5	1338.8		

## Data Availability

All relevant data are provided within the paper and no additional data are available.
